# Measuring Phases of Employment Decision-Making and the Need for Vocational Services as a Social Determinant of the Health of Employed People Living with HIV

**DOI:** 10.3390/ijerph192215074

**Published:** 2022-11-16

**Authors:** KB Boomer, Liza M. Conyers, Yili Wang, Yung-Chen Jen Chiu

**Affiliations:** 1 Department of Mathematics, Bucknell University, Lewisburg, PA 17837, USA; 2 Department of Counselor Education, Counseling Psychology, and Rehabilitation Services, Pennsylvania State University, University Park, PA 16802, USA; 3 Department of Biostatistics, University of Michigan, Ann Arbor, MI 48109, USA; 4 Department of Educational Foundations and Counseling, Hunter College, City University of New York, New York, NY 10065, USA

**Keywords:** HIV, social determinant of health, employment, work

## Abstract

(1) Background: Secure employment has been recognized as a social determinant of health for people living with HIV (PLHIV), but limited research has been conducted to understand the employment needs and vocational decision-making process of those who are employed. The purpose of this study is to examine the applicability of the client-focused considering-work model to assess the employment outcomes and employment decision-making phases of a sample of employed PLHIV. (2) Methods: This study analyzed data of 244 employed PLHIV who completed National Working Positive Coalition’s Employment Needs Survey which included a 20-item Considering Work Scale-Employed version (CWS-Employed) and a single-item Classification of Employment Status Scale (CESS). Factor analysis was used to evaluate the CWS-Employed. Chi-square tests of homogeneity of proportions were conducted to assess the domain-specific needs of individuals in each phase of employment decision-making. (3) Results: Our findings revealed high rates of insecure employment and diverse vocational service needs among research participants. Additionally, the CWS-Employed accurately predicted 71% of the self-reported classification of phases of employment decision-making. (4) Conclusions: When investigating the role of employment as a social determinant of health, more research is needed to better understand the vocational needs and outcomes of PLHIV who are working. Improving the measurement of the phases of employment decision-making is needed to better identify appropriate vocational interventions that can lead to improved employment and related health outcomes for this population.

## 1. Introduction

While many recognize the benefits of employment as a social determinant of health for people living with HIV (PLHIV) [1,2], little attention has been devoted to understanding the vocational service needs of PLHIV who are employed. Evaluating work options and vocational needs after a diagnosis of HIV often requires an assessment of multiple interrelated factors including medical, psychosocial, financial, legal, and vocational [3]. As such, it is not surprising that individuals with HIV and other episodic illnesses or disabilities face many challenges when trying to determine what vocational decisions would be best in the face of uncertain health [4,5]. Receiving an HIV diagnosis can be traumatic and disruptive to employment, as individuals process the medical and psychosocial implications of living with HIV. Many are not aware of legal rights for people with disabilities/chronic health conditions or resources that can help them to maintain work despite any physical or mental health concerns. People who are employed when diagnosed with HIV or other chronic illnesses must both assess if they have the capacity to continue to perform the essential functions of their job with or without accommodation, and determine the impact of work demands on their long-term health, including their ability to adhere to critical treatment protocols. This assessment is far more complicated when one is faced with an episodic illness [6]. While several efforts have been devoted to addressing and assessing the return-to-work process for people with work-related injuries [7,8], psychiatric disability [9], or those participating in vocational rehabilitation services [10], little research addresses the decision-making process regarding remaining at work for those living with episodic illness, including HIV. Although some evidence supports the benefits of vocational rehabilitation on increasing access to care and reducing health risk behaviors for PLHIV, no studies have explored the relationship between vocational decision-making on HIV health and prevention among employed PLHIV

To advance research in this area, Conyers and Boomer [11] adapted the client-focused considering-work model for PLHIV (CWMHIV) and proposed a revised considering-work model for people with emergent or episodic illnesses (CWMEE [12]). Key aspects of the original CWMHIV were retained, including placing primary value on client-focused decision-making and recognition of the non-linear nature of the decision-making process. Both the original and revised considering-work models (CWM) posit that transitions in employment status are first considered when there is pressure to change and that medical, psychosocial, financial/legal, and/or vocational domains of influence facilitate and/or hinder transitions into and/or out of employment. An innovative aspect of the CWM framework is the adaptation of the transtheoretical model of change [13] applied to four phases of considering a change in employment status: contemplation, preparation, action, and resolution. Rather than promoting change in employment status as a desired outcome, the primary focus of the CWM framework is to provide a structure for clients and providers to help guide vocational decision-making and service provision that considers medical, financial/legal, psychosocial, and vocational domains of influence in the decision-making process. As such, the definitions of the phases of change are not defined by expectations to implement changes within a six-month timeframe; rather, the CWMEE phases provide a pathway for clients and providers to conceptualize engagement in vocational services in a more holistic way. To better distinguish each of these phases and to clearly measure transitions into and out of paid employment, the CWMEE revised the definitions of the preparation and action phases as originally proposed in the CWMHIV. Unlike the original CWM, the CWMEE also distinguishes between secure and insecure employment outcomes. Although these phases of change and outcomes can help to guide and evaluate phase-based employment interventions, they are not theorized to be linear. Consistent with the client-focused approach, individuals may move back and forth between phases and outcomes as they obtain more information or as circumstances change. As noted by the inclusion of the “client-focused” in the title of this model, the primary focus of the vocational decision-making process is the overall health and well-being of the client, not their ultimate employment status.

**Figure 1:**
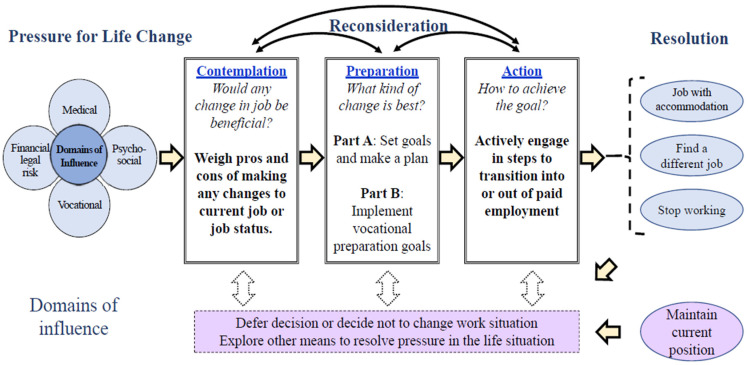
The Considering Work Model for Employed People with Chronic Illness identified the five phases of contemplation, preparation—set goals and make a plan, preparation—implement goals, action, and resolution.

To date, the CWM framework has primarily been applied to conceptualizing transitions from not working to becoming employed with either secure or insecure employment outcomes. However, as noted in Figure 1 below, the CWMEE can also be applied to people with HIV or other chronic illness who are employed and includes a wider range of decisions related to changing current job conditions (i.e., job accommodation), finding a different job, deciding to stop working for pay, or making no changes.

Within the CWMEE—Employed framework, the primary task of the contemplation phase is to determine *if any change in current employment would be beneficial.* In this phase, individuals who are currently employed focus on evaluating the pros and cons of remaining in their current position and/or changing their working conditions (e.g., change in hours, transition to a different position, requesting accommodations, transition to unemployment/retirement). The main task of the preparation phase is to *consider which change is best*. In this phase, individuals develop a plan to evaluate which change in employment status would be best and then take the steps needed (e.g., career exploration, retraining, benefits counseling) to help prepare them to make desired changes to their current job or job status. The action phase focuses on *how to implement the desired change* and includes tasks designed to address the pressure to change (e.g., requesting a job accommodation, applying for a different job(s), going on job interviews) and/or terminating their current position (e.g., submitting a letter of resignation/retirement). In the resolution phase, individuals assess whether the pressure to change jobs has been resolved. The resolution phase could include a decision not to make a change in one’s job if the pressure to change was resolved through other means.

The ability to classify phases of considering work for PLHIV has several advantages. From a service-delivery perspective, identification of the phase of considering work could help to guide the selection of phase-based vocational interventions. For example, someone who could benefit from adapting their current job requirements/conditions may need to evaluate the pros and cons of making these changes and may need benefits counseling and/or consultation regarding their legal right to a job accommodation. In the preparation phase, a person may need training on how to request accommodation, skills training to be eligible for a different type of job, and/or job-seeking training. Likewise, someone in the action phase may need assistance with identifying and applying for specific jobs. Research indicates that many PLHIV underutilize vocational rehabilitation resources [11,14], which may be a consequence of a lack of knowledge of or access to these services and not having a framework to help guide their employment decision-making process. Knowledge of a person’s phase of considering work could help determine what type of information and potential referrals for vocational services would be most relevant to advancing economic opportunities for those who otherwise do not have access to this information, while also considering the impact of employment on health and psychosocial needs. Unlike other vocational rehabilitation models that tend to focus primarily on employment outcomes, the CWM approach includes broader domains of influence on vocational decision-making, including medical, financial/legal, psychosocial, and vocational.

The considering-work framework has been used to inform the development of vocational services for PLHIV, such as the Foundations for Living Program [15] and the Common Threads Program [16], and staff development training (e.g., Getting to Work [17]). Nevertheless, no research has been carried out to explore the application of the considering-work model for PLHIV who are employed or to consider how this model can inform the role of employment and vocational decisions as important social determinants of HIV care and prevention. Within this context, the objectives of the current study are to use the data from the 2018–2019 National Working Positive Coalition’s Employment Needs Survey to (a) describe key job characteristics including the level of job security among a diverse sample of employed PLHIV, (b) explore the measurement of phases (i.e., contemplation, preparation, action) of considering changes to respondents’ current employment, and (c) explore the relationship between the impact of employment decision-making and social determinants of HIV health.

## 2. Methods

### 2.1. Survey Research

This study is based on participant data from the 2018–2019 National Working Positive Coalition (NWPC) Vocational Development and Employment Needs survey. The research participants for this study were recruited from HIV Service Organizations and networks across the U.S. with targeted recruitment in New York. An IRB-approved recruitment email was sent to HIV network listservs and HIV service organizations in various states in the U.S. Individuals who had some level of contact or affiliation with HIV service organizations, HIV support groups, and networks of PLHIV received the recruitment emails. To protect the privacy of individuals, the recruitment emails were sent directly to the individuals by the coordinator of the listservs or HIV service-organization directors. Criteria for study inclusion were being a United States citizen or permanent resident, 18 years of age or older, and living with HIV. Participants completed the National Working Positive Coalition’s Employment Needs Survey, which contained over 100 questions, including the 20-item Considering Work Scale—Employed Version. All responses were anonymous. Respondents completed the online survey in an average of 32.7 min and received a USD 15 e-gift card for their participation. The data were collected from December 2018 through September 2019.

### 2.2. Participants

This study uses the responses of 244 employed participants who recorded complete responses for the 20 Considering Work Scale-Employed version items. The participants represented diverse racial (44% Black, 41% White, 6% Multiracial, 4% Native American, 3% Asian, 2% Native Hawaiian/Pacific Islander), gender (57% male, 43% female), ethnicity (42% Latino/a/x), and sexual-orientation (55% heterosexual, 34% gay, and 11% bisexual) backgrounds. Within the sample, 42% live in the Northeast, 29% live in the South, 17% live in the West, and 12% live in the Midwest; a total of 34% of the sample is from New York State. The average reported age is 39 years old, with a minimum of 23 and a maximum of 71 years old.

### 2.3. Instruments

This study used two measures, a single-item self-report Classification of Employment Status Scale (CESS) and the Considering Work Scale-Employed (CWS-Employed). These measures were developed based on the stages of change theory, which has been adapted in the field of career counseling and vocational rehabilitation. The original stage of change model [13] has five stages, including pre-contemplation, contemplation, preparation, action, and resolution/maintenance. Other measurements based on the stages of change theory (i.e., Stage of Change for Employment Scale (SOCES-12) [18], the University of Rhode Island Change Assessment for Vocational Counseling [URI-CA-VC] [19], the Lam Assessment on Stages of Employment Readiness [LASER] [20], and the modified Stage of Change Survey [21]) were designed to address vocational decision-making for unemployed individuals and incorporated varied definitions for differing phases adapted from stage of change theory.

The CWM framework does not include a pre-contemplation recognizing that not working is not inherently problematic for individuals with HIV or other chronic illnesses. Although the CWMEE-Employed model only has four phases of considering work, including contemplation, developing a plan, preparation, action, and resolution, the preparation phase was theorized to have two stages. The single-item CESS consisted of descriptions of each of the CWMEE-Employed phases of considering work, including the subsections of the Preparation phase. To self-classify, respondents were asked to “select the option below that best describes your current thoughts and plans about employment,” based on the descriptions in Table 1.

The 20-item CWS-Employed Version was developed to measure the different phases of considering work based on the CWMEE model. Items are listed in Table 2.

### 2.4. Data Analysis

Statistical analyses were conducted using Mplus v7.4 (Los Angeles, CA, USA) and SAS v9 (Cary, NC, USA). Descriptive statistics were used to assess the percent of individuals who reported secure or insecure employment as well as other employment outcomes (e.g., number of jobs). Chi-square tests of homogeneity of proportions were conducted to assess the domain-specific needs of individuals in each phase of considering work. Factor analysis was used to evaluate the CWS-Employed. As each of the 20 considering-work items was recorded on a five-point Likert scale (1 = strongly disagree to 5 = strongly agree), analyses treated the items as ordinal categorical variables. An exploratory factor analysis was conducted to assess how the 20 items contributed to the proposed decision-making phases. An ordinal logistic regression analysis was conducted to assess the agreement between the factor structure and the self-reported phase of change in employment status.

## 3. Results

Eight percent of the respondents had two or more jobs. While 44% were salaried employees and 33% were hourly wage employees, 16% were paid by a stipend, 6% were self-employed, and 1% reported informal work. Nearly 52% reported having secure employment while 48% reported being uncertain whether they could keep or maintain their current job. There is not a significant association between employment security and employment payment (i.e., salaried versus hourly wage, chi-square 3.11, df = 3, *p*-value 0.3749, excluding the informal-work group).

The measurement of the considering-work phases (e.g., contemplation, action) began with an exploratory factor analysis of the considering-work scale. This was suggested based on an analysis of the interrelatedness of the 20 items. Each of the 20 items had a polychoric correlation of at least 0.4 with at least four other items, with the exception of CW4 (“I have the ability to maintain my job and stay at in my current position”) which has one pairwise correlation greater than 0.4 and four correlations greater than 0.35. The minimum communality among the items was 0.244; excluding CW4, the minimum communality was 0.441. Thus, each item shared some common variance with the other items. The Kaiser–Meyer–Olkin measure was 0.88, indicating a factor analysis is appropriate [22,23].

An exploratory factor analysis was conducted using a weighted least squares approach which treated each of the items as an ordinal variable [24]. Based on the initial correlation analysis, CW4 was removed from the analysis. Analysis was conducted using four oblique rotations (promax, CF-varimax, CF-equamax, and oblimin) to minimize the correlation among the resulting factors. Factor loadings of 0.4 or greater were retained; the four rotations produced similar factor-loading results. The CF-varimax and CF-equamax yielded the smallest standard errors of the rotated loading, with the same minimum of 0.032 and similar maximums of 0.080 (CF-equamax) and 0.085 (CF-varimax). The CF-equamax results reported as only one item had a cross-loading exceeding 0.4 (CW9 with the preparation factor). The comparative fit index (CFI) was 0.96 and the Tucker–Lewis Index (TLI) was 0.932, indicating a good to very good fit. The root mean square error of approximate (RMSEA) was 0.072, less than 0.1, indicating a good fit.

Initially, it was theorized that developing a plan was the first part of the preparation phase (Table 3). However, the contemplation and developing-a-plan items loaded together onto a single contemplation factor with the part-B-of-preparation (implementation of preparation items) loading as a second factor. Therefore, a four-factor model was fit incorporating the developing-a-plan items into the contemplation phase of considering work. There is some suggestion that CW9 (“I am making contacts to get interviews for a different job”) loaded onto both the preparation factor and the action factor with respective loadings of 0.426 and 0.502.

The consistency of the items associated with each of the four factors was assessed with an ordinal measure of Cronbach’s alpha [25,26] with the following results: contemplation and developing a plan = 0.87, preparation = 0.77, action = 0.86, and resolution = 0.85.

Considering-work factor scores for the four phases were computed as the average of the items identified for each factor. One of the study participants reported the same score on each of the four factor scores, which was viewed as suspect and was further removed from the analysis. The considering-work factor scores were approximately symmetric, covered the possible scale of 1 to 5, had similar variability, and were centered near the middle of the items’ five-point scale (Table 4). The contemplation/developing a plan, preparation, and action latent variables were negatively associated with the resolution phase and positively associated with each other. The variance inflation factor (VIF) for each variable was assessed in a linear regression model and found to range from 1.28 to 1.86; as these VIF values are less than 5, no multicollinearity is expected in the ordinal logistic regression. Thus, these factor scores are assumed to have equal variance and be independent.

An ordinal logistic regression on the remaining 242 data observations was conducted to assess the agreement between the considering-work factor scores and the self-categorization into different phases of considering work (the outcome variable). The model classified respondents into the four phases based on the highest predicted probability of group membership. The concordance between the scale-based predicted phase group membership and the self-categorization phase was found to be 71.1%. Thus, 71.1% of the time, the considering-work scale categorized an individual into their self-reported phase, on average.

In the sample, 18.5% of the respondents self-categorized as being in the contemplation phase and 28.8% as being in the developing a plan phase. To be consistent with the outcomes from the factor analysis, these two groups were combined into a single group representing the combined phase (*n* = 115, 47.3%). A total of 11.1% (*n* = 27) were in the preparation phase, 6.6% (*n* = 16) were in the action phase, and 35% (*n* = 85) were in the resolution phase. One of the study participants did not report a phase and was removed from the ordinal logistic regression analysis.

The association between key medical, psychosocial, financial/legal, and vocational domains of influence across the four phases in which the respondents self-categorized are summarized in Table 5. Reported outcomes were found to be significantly associated with the phases of work with chi-square tests of homogeneity of proportions. Looking at the medical-domain items, we found that although most respondents report a decrease in viral load since starting to work (56.25% to 89.5%) regardless of employment decision-making phase, high proportions of respondents (37.5 to 81.6%) may stop working due to unstable health. Within the psychosocial domain, high proportions of respondents across the phases of considering-work report both a decrease in health-risk behaviors (66.7% to 87.5%) and a decrease in drug and/or alcohol use (65.9% to 96.2%). Results of the financial domain indicate that those within the action phase have the lowest proportion of access to health insurance through their job (45.5% compared to 82.3% or higher in other phases). Within the vocational domain, those in the contemplation (55.2%) and preparation (55.6%) phases report the highest proportion of need for a job accommodation, while those who are resolved not to make a change at work report a relatively low level of need for accommodation (12.3%). The two most needed accommodations across all employment decision phases are to change work schedule and to be able to take medications, with those in the action phase identifying change in work schedule as the greatest need (83.3%) and those in the preparation phase most needing time to take their medications.

## 4. Discussion

The purpose of this paper was to explore the application of the CWMEE to employed individuals living with HIV. To examine the need for a considering-work model for employed individuals, we first described perceptions of job security among a diverse sample of individuals who completed the National Working Positive Coalition’s Employment Needs Survey. We then explored the validity of the CWS-Employed, which was designed to measure employment decision-making phases. Finally, we examined the association between the phases of employment decision-making and HIV health and vocational service needs. In response to these goals, this study makes several contributions to the research literature. First, this study quantifies the high levels of insecure employment reported by a diverse sample of employed PLHIV, supporting the need for vocational rehabilitation support post-employment. Second, the study found support for the ability of the CWS-Employed to measure employment decision-making phases. Finally, the study identified important associations between the phases of employment decision-making and the HIV health and vocational needs of employed PLHIV. Overall, the findings support the hypothesis that employed PLHIV experience high levels of insecure employment and have diverse health and vocational service needs. Although the application of the CWMEE to employed individuals with HIV, an episodic illness, can be useful to inform service delivery and future research, more research is needed to refine the CWS-Employed to continue to examine the role of employment as a social determinant of HIV health and prevention.

### 4.1. Precarious Employment among People Living with HIV

According to the World Health Organization (WHO) Commission on Social Determinants of Health [27], employment and working conditions affect health and health equity, as they lead to other factors important to health including “financial security, social status, personal development, social relations, self-esteem, and protection from physical and psychosocial hazards” (p. 72). The findings from this study indicate that there are high levels of insecure employment among the respondents, with 48% reporting that they are uncertain whether they could keep or maintain their current job. Additionally, eight percent of the sample reported having two or more jobs, suggesting that one job did not provide enough income or security to suffice. Although existing biomedical interventions provide a pathway to ending the HIV epidemic, unemployment, insecure work, and/or precarious work conditions can interfere with positive HIV health and prevention outcomes, perpetuating economic and health disparities. Studies that examined PLHIV’s labor-market experiences found that employment is associated with optimal physical-health and mental-health status, and a higher level of quality of life [28]. Nevertheless, Rueda et al. [29] found that adverse psychosocial work conditions (i.e., psychological demands, job insecurity, and low decision authority) are associated with depressive symptoms that are similar to unemployment. Therefore, insecure work or adverse working conditions may have no benefit compared to being unemployed. Populations most impacted by HIV not only experience higher levels of employment discrimination and unstable work, but they also face disparate access to vocational services that could help prepare them for better quality jobs and/or help them to maintain employment in the face of health or other challenges threatening loss of employment [16].

Of note is that health status can be a determinant of employment and workforce participation for people with chronic health conditions. For example, literature on cancer survivorship and multiple sclerosis has identified a relationship between health status and unemployment, underemployment, and precarious work conditions [30]. Mehnert [31] found that cancer survivors are more likely to experience changes in work schedules, wages, and work environments compared to those who do not have cancer. Physical and mental limitations, the impact of treatment needs on work schedules, job discrimination, job accommodations, and continuity of care are factors that may influence employment decisions for individuals with chronic illness [32]. Other social determinants of health such as age, gender, race/ethnicity, and symptom severity play an important role in employment decisions as well [31]. Our findings related to the high rates of insecure work among employed PLHIV underscore the need for vocational rehabilitation services that can better evaluate the intersection of health and work and integrate access to resources and accommodations that are known to improve employment outcomes for people with chronic health conditions. Despite the research indicating that better quality employment is associated with higher levels of quality of life, employment-related research has not been prioritized for PLHIV. The inclusion of a focus on quality of life of PLHIV in the most recent National HIV/AIDS Strategic Plan 2022 to 2025 [33] underscores the need for more research in this area, including increasing the capacity to assess the vocational development needs and decision-making process of employed PLHIV.

### 4.2. Measurement of Vocational Decision-Making among Employed PLHIV

While some research has focused on assessing the vocational rehabilitation and employment decision-making needs of unemployed PLHIV [34,35], limited attention has been devoted to these needs among those who are employed. To address this need, our study evaluated the CWS-Employed, which was developed to assess phases of vocational decision-making among employed PLHIV based upon the CWMEE-Employed. Although the CWMEE-Employed theorized a five-factor model (including two factors within the preparation phase), our results identified a four-factor model. This outcome is consistent with previous research that identified three to four factors with varied stages of change scales developed for people with a range of disabilities who were unemployed [19,20,36].

Although these findings provide support for the existence of varied phases of decision-making among employed PLHIV, they also indicate that revisions to the model are needed. The proposed “developing a plan” items that were theorized to be part one of the preparation phase aligned with the contemplation phase. Upon closer scrutiny of these items, the researchers recommend several refinements to the model and scale. For one, many of the items related to developing a plan were too broad with a lack of a specific focus on plan development, which helps to explain why these items tended to load on the contemplation phase. Furthermore, given that the need to develop a vocational plan could apply to each phase of the employment decision-making, we recommend that the construct of plan development be removed from this scale.

To further examine the validity of the CWS-Employed, we assessed the probability that this scale could predict respondents’ self-reported phase of employment decision-making as measured by the single-item Employment Decision-Making Scale (EDMS). Our findings suggest that the CWS-Employed accurately predicted 71% of the self-reported phases of employment decision-making changes, supporting both the usefulness of the scale and the need for further research. As with the CWS-Employed, further refinement of the EDMS is needed to remove the construct of developing a plan from this measure. Future research could also incorporate other measures related to task-specific self-efficacy (e.g., job seeking self-efficacy) and/or measures of outcome expectations (e.g., availability of other jobs in my field) to further validate the CWS-Employed. In light of this study’s outcomes, the authors have revised the EDMS scale with the new items presented in Table 6.

### 4.3. Exploring the Relationships between Domains of Influence and Employment Decision Phases

The finding that respondents self-classified across all phases of employment decision-making provides support for the variability of vocational rehabilitation needs among employed PLHIV. About 20% reported being in the contemplation phase and were characterized by positive health changes (decrease in health-risk behaviors and decreased alcohol and/or (non-injection) drug use) and a moderate need for workplace accommodation. These findings suggest that changes in health behaviors can lead to an interest in changing jobs and/or need for work accommodation assessment. Those in this phase of employment decision-making have not yet decided to make a change and may need more information and opportunities to evaluate the pros and cons of making a change to determine the next steps. However, prior research indicates that many PLHIV do not have knowledge of the vocational services that could help inform their decisions [34].

The largest proportion of respondents reported being in the preparation phase (40%), of which about 29% self-classified as developing a plan while 11% reported that they were preparing to make a change. Similar to those in the contemplation phase, a high proportion of those in the preparation phase also reported improved HIV health outcomes and reduction in alcohol and/or drug use since being employed. However, this group has already decided that they want to make a change, which would suggest a potential need for different types of vocational services. Although some people with episodic disabilities may be able to change jobs without preparation, given the complexity of issues associated with episodic and chronic illness, having support to become fully aware of available resources and to evaluate goals is highly recommended [12]. According to Goldblum and Kohlenberg [3], developing effective goals can be time-consuming and takes careful consideration not to be too broad or too specific, as neither extreme is helpful. Seeking consultation from disability-law, vocational-rehabilitation, and financial-planning experts can help to support well-informed decision-making during this process. Importantly, state vocational rehabilitation counselors can provide services to help individuals with disabilities to maintain their job, particularly when a potential loss of employment is health-related.

That only six percent of respondents self-classified as being in the action phase despite the high rates of insecure work reported by the overall sample reflects the multiple barriers that PLHIV likely experience when trying to find better quality employment. The majority of those in the action phase reported unstable health (71.4%), with only 45.5% having access to health insurance through their jobs. This finding underscores the importance of evaluating the quality of employment when providing vocational rehabilitation or employment services to this population. Given the importance of access to health care, it makes sense that these individuals would be motivated to change jobs. Although a high proportion of those who are resolved not to change their employment status also report unstable health (81.6%), the vast majority in this phase (85.9%) also report having access to health insurance through their job, which could be a key factor in choosing to remain employed.

The finding that many respondents also reported that they may stop working due to unstable health highlights the challenge that many people with episodic illness face when contending with uncertain health outcomes. Prior research investigating employment as a social determinant of health has stressed the value of vocational rehabilitation services to HIV care and prevention. Medical advancements in HIV treatment and outcomes have led to greater restrictions on public-health benefits such as social security and many PLHIV have to work to survive. Research findings indicate that the use of vocational rehabilitation services is associated with reduced health-risk behaviors and increased access to care for PLHIV [34]. Additional research indicates that having a strong vocational identity is associated with a reduction in problem behavior, as well as improved mental health and well-being [28,37]. Furthermore, individuals who report higher levels of job-seeking self-efficacy report being less impacted by their chronic health conditions. However, disparate access to employment services and resources designed to maintain and/or advance the quality of employment of PLHIV impacts the employment decision-making process and may lead to premature separation from employment that contributes to the high rates of unemployment (41%) of PLHIV [38].

Considering the role of employment as a social determinant of health, it is important to note that the majority of respondents, regardless of their phase of employment decision-making, reported a decrease in viral load, health risk behaviors, and drug/alcohol use since starting to work. This finding is consistent with other studies that have found support for the role that employment can play as a positive social determinant of health [2,39]. In a meta-analysis of research investigating the association between employment status and adherence to HIV medications, Nachega et al. [40] suggest that employed individuals were 27% more likely than unemployed individuals living with HIV to adhere to antiretroviral medications. Others have reported on both the physical- and mental-health benefits of employment for many but not all PLHIV [41]. One study, for example, found that being employed was associated with loss of HIV care [42]. Some research has explained these discrepancies as being related to the complex management of both HIV and earning a living [43]. In sum, when considering the impact of employment as a social determinant of health and prevention, it is critical to evaluate the quality of employment. Unlike other models of vocational decision-making, the CWMEE-Employed does not assume that being employed is the best outcome, as it underscores the importance of examining additional financial/legal, psychosocial, medical, and vocational domains of influence on the employment decision-making process.

### 4.4. Limitations and Future Research

This study has some limitations. First, we used a volunteer sample. Those who choose to participate in the study may not represent the PLHIV who are currently working. A second limitation of this work is the relatively small sample size (*n* = 242). Only 16 individuals (6.6%) reported being in the action phase. This is a limitation in the statistical sense as it limits the potential to draw inferences from the larger population. Given that the survey was conducted online, individuals had to use technology to complete the questionnaire. Therefore, we might lose individuals who had difficulties accessing technology or were not competent to complete a survey online. Replication of the current study with a larger and more diverse sample would help better understand the phases of employment decision-making among PLHIV and continue to develop the instrument. Experimental studies can also be applied to control individuals in different phases to obtain a more balanced sample. Exploring the associations between phases of employment decision-making and employment outcomes among PLHIV should be considered in future research. Employment outcomes can be tracked longitudinally to understand the changes in the phases of employment decision-making.

## 5. Conclusions

Community input and the government Ending the HIV Epidemic and national, state, and local HIV/AIDS strategy plans identify employment and economic needs as key drivers of racial, ethnic, gender, and health disparities in HIV care and prevention outcomes. Although work plays a primary role in life, frequently providing access to income, social support, a sense of purpose, and other factors related to physical and mental well-being, efforts to measure employment decision-making and examine employment as a social determinant of HIV health have been minimal. Since research indicates that vocational development, use of vocational rehabilitation services, and employment status are associated with HIV health and prevention outcomes, having an instrument that could assess phases of employment decision-making and employment status over time could be instrumental in our ability to better assess the impact of employment as a social determinant of HIV health and prevention. Vocational rehabilitation services and workforce development programs need to first assess individuals’ current phase of employment decision-making as well as the medical, psychosocial, financial/legal, and vocational domains of influence that can impact and be impacted by employment decisions. Matching services with the levels of readiness of recipients may also be a cost-effective strategy for service providers, as they can use the resources more efficiently [20] given that prioritized vocational services or resources are needed to help individuals at different phases.

While HIV service providers have developed a robust system for addressing the primary health and prevention needs of people living with or with greater vulnerability to HIV, few are trained to assess or respond to employment needs. Medical case managers and rehabilitation professionals can apply the CWMEE-Employed to better understand the complex needs of PLHIV. Psychosocial education, benefits counseling, and referral to social services (transportation, child-care services) are all strategies that could better inform employment decisions. In helping individuals who are ambivalent and reluctant to change, Motivational Interviewing (MI) [44] is an evidence-based approach to help individuals to process ambivalence and make decisions best suited to their individual circumstances. Rehabilitation professionals can apply MI to help individuals identify and explore employment goals, including consideration of the impact of employment on their overall health and well-being. Strategies including confidence ruler, cost-benefit analysis, and roll with resistance can be used to motivate individuals to move from one phase to another, as appropriate. Rehabilitation professionals also need to recognize some individuals are satisfied with their current work conditions and do not want to change. A discussion on how to maintain their employment and work–life balance can be included in the services. Given the research that indicates the associations between the quality of work and health outcomes, more resources are needed to improve the quality of employment. For example, using Goldblum and Kohlenberg’s research [3], the U.S. Department of Labor has developed a Toolkit [45] that encourages PLHIV to consider their individual strengths and barriers within each phase to help make informed career decisions.

On a systems level, the limited engagement of the Department of Labor or the Department of Education in the development and implementation of the National HIV/AIDS Strategy has also resulted in limited to no additional service responses tailored to the employment needs of PLHIV. In light of the increased focus on employment as a social determinant of health in state- and national-level HIV strategic planning, there is a need for greater levels of cross-system collaboration across government and community-based health, workforce development, education, housing, legal, and other service systems to reduce systemic barriers to employment for people living with HIV.

## Figures and Tables

**Table 1:** Classification of Employment Status Scale (CESS).

Employment Decision-Making Phase	Description Used as a Prompt for Respondents
Contemplation	Contemplating pros and cons of changing jobs or position—I think about changing my job from time to time, but I have not decided whether changing my job or looking for a different position is a good option for me right now.
Preparation A: Developing a Plan	Developing a plan—I know that I want to change jobs, but I need more time to explore what other work options are best for me, and to identify what I would need to do to get ready for a change.
Preparation B: Preparing to achieve plan goals	Preparation—I know what I need to do to achieve my vocational goals, and I am now taking the steps needed to prepare for a change before I am ready to start applying for different jobs.
Action	Action—I am applying for different jobs (sending out applications/resumes, going on job interviews, etc.).
Resolution	Not considering a change in jobs—I am not thinking about changing my job at this time.

**Table 2:** Considering Work Scale-Employed (CWS-Employed).

Considering Work Phase	Item
Contemplation	5. I would like to have some advice about whether changing jobs is a good idea for me.
	12. I think about getting a different job sometimes but I am afraid it may not be good for me.
	17. Having a different job might be a positive change for me but I am not sure.
	20. I am debating the pros and cons of whether changing my job is a good idea for me right now.
Developing a Plan	1. I want to change jobs but I need to explore what options would be best for me before I start looking for a different job.
	7. I need to figure out my employment goals and needs before changing my job.
	14. I wish I knew what I need to do to get a different job that I would like better.
Preparation	3. I am taking the steps I need to prepare for the type of work I would like to do in the future.
	8. I am currently participating in job training or other activities so I will be better prepared to get a different job.
	11. I am completing the steps I need to take to become more prepared before I start to look for a new job.
	19. For now, I am focused on getting the education/training that I need to get the type of job I want in the future.
Action	13. I am doing everything I can to find a new job within the next six months.
	16. I am applying for a different job.
	6. I am actively looking for a different job.
	9. I am making contacts to get interviews for a different job.
Resolution	10. I plan to keep my current job
	15. My current job meets my needs
	18. I don’t have any plans to change jobs
	2. I am satisfied in my current job
	4. I have the ability to maintain my job and stay at in my current position.

**Table 3:** Four-factor solution using the CF-Equamax rotation.

Hypothesized Considering Work Phase	Item	Factor 1: Preparation	Factor 2: Contemplation and Developing a Plan	Factor 3: Action	Factor 4: Resolution
Contemplation	CW5	−0.102	**0.686**	0.231	0.081
	CW12	0.181	**0.714**	−0.125	0.038
	CW17	0.099	**0.652**	0.133	−0.117
	CW20	0.105	**0.598**	0.280	0.133
Preparation Part A: Developing a plan	CW1	0.167	**0.512**	0.172	−0.213
	CW7	0.280	**0.654**	−0.191	−0.115
	CW14	−0.033	**0.475**	0.352	−0.096
Preparation Part B: Implementing preparation goals.	CW3	**0.669**	0.064	0.018	−0.112
	CW8	**0.585**	−0.121	0.329	0.203
	CW11	**0.498**	0.207	0.150	−0.146
	CW19	**0.692**	0.050	0.020	0.121
Action	CW6	0.139	0.086	**0.606**	−0.291
	CW9	0.426	−0.060	**0.502**	0.006
	CW13	0.198	−0.031	**0.700**	−0.004
	CW16	0.044	0.064	**0.724**	−0.226
Resolution	CW2	0.220	−0.002	−0.240	**0.719**
	CW10	−0.122	0.068	−0.033	**0.760**
	CW15	0.085	−0.012	−0.106	**0.811**
	CW18	−0.172	−0.055	0.041	**0.642**

**Note:** Numbers in bold indicate the largest loading for each survey question.

**Table 4:** Descriptive statistics for the CW factor scores (*n* = 242) created as the average of items on a five-point Likert scale.

Factor Score	Mean	Standard Deviation	Minimum	Maximum
Contemplation/developing a plan	3.24	0.827	1.0	4.9
Preparation	3.20	0.831	1.0	5.0
Action	2.80	0.891	1.0	5.0
Resolution	3.52	0.805	1.0	5.0

**Table 5:** Relationships between domains of influence and employment decision phase.

Domain	Item	Contemplate/Plan	Preparation	Action	Resolve
Medical	May stop working due to unstable health ^[a]^	56.0%	37.5%	71.4%	81.6%
	Since starting work, viral load has decreased ^[b]^	69.8%	88.9%	56.25%	57.7%
Psychosocial	Since starting work, health risk behaviors decreased (*p* = 0.059)	81.0%	66.7%	87.5%	67.1%
	Drug and/or alcohol use decreased ^[a]^	85.3%	96.2%	75%	65.9%
Financial	Has health insurance through job(s) ^[a]^	82.3%	95.5%	45.5%	85.9%
Vocational	Need an accommodation ^[a]^	55.2%	55.6%	31.3%	12.8%
	Accommodation needed: Change in work schedule ^[a]^	24.1%	12.5%	83.3%	29.2%
	Accommodation needed: Allow time to take medicine ^[a]^	50.6%	93.75%	50%	41.7%

Notes: ^[a]^ significant at 0.01; ^[b]^ significant at 0.05.

**Table 6:** Employment Decision-Making Scale-Employed-Revised (EDMS–Employed-Revised).

Employment Status phase	Description Used as a Prompt for Respondents
Contemplation	I am considering the pros and cons of changing my job or staying at my current job/position.
Preparation	I am working toward my goals to be better prepared before applying for jobs (e.g., job exploration, education, training, counseling).
Action	I am applying for different jobs (e.g., sending out applications/resumes, going on job interviews) or requesting a change to my current position (e.g., change in work schedule, reducing work hours).
Resolution	I do not want to change my job/position at this time.

## Data Availability

Not applicable.
